# Behavior of CFRP-Confined Sand-Based Material Columns under Axial Compression

**DOI:** 10.3390/polym13223994

**Published:** 2021-11-19

**Authors:** Guodong Li, Honglin Liu, Wentao Deng, Hongzhi Wang, Haitian Yan

**Affiliations:** 1 College of Geology and Mining Engineering, Xinjiang University, Urumqi 830046, China; cklgd2011@xju.edu.cn (G.L.); dddwt661@163.com (W.D.); cumterwhz@163.com (H.W.); yht@stu.xju.edu.cn (H.Y.); 2 Key Laboratory of Environmental Protection Mining for Mineral Resourses at Universities of Education Department of Xinjiang Uygur Autonomous Region, Xinjiang University, Urumqi 830046, China

**Keywords:** desert sand, high-water material, fiber-reinforced polymer, composite structure, underground mines

## Abstract

This paper presents an innovative pumpable standing support designed for underground mines located in the arid and semi-arid deserts of the Gobi region with a shortage of water resources. The exterior shell of this pumpable standing support is made of carbon fiber-reinforced polymer (CFRP), while the infill material is a sand-based material (SBM). As the novel backfill material, SBM is the combination of high-water cementing material and desert sand. A series of experimental tests were conducted to obtain the mechanical response mechanism of this novel pumpable standing support under uniaxial compression. Test variables investigated in this research covered the water-to-powder ratio of the cementing material, the mixing amount of sand, and the thickness of the CFRP tube. Test results confirmed that the CFRP-confined SBM columns exhibited typical strain hardening behavior with the acceptable axial deformation. It was also demonstrated that using high-strength cementing material, a thicker CFRP tube, and a high mixing amount of sand effectively increased the bearing capacity of the CFRP-confined SBM column. Except for the exemplary structural behavior, the consumption of high-water cementing materials of the novel pumpable standing support is smaller than that of its counterparts made of pure cementing material, when specimens with the same mechanical performance are compared.

## 1. Introduction

Increasingly complex production demands and challenging geological conditions in underground mining have severely impacted the stability control of roadways surrounding rocks [1]. Therefore, the bolting supports alone can hardly adapt to the highly diversified geological conditions. The combination of active and passive supports for surrounding rock control in the roadway is of high importance for ensuring the stability of surrounding rocks in underground mines [2,3]. At present, passive supports (also known as the auxiliary support system) are commonly used for the stability control of roadways surrounding rocks in underground mining, mainly including timber piles, prop walls, concrete blocks, and reinforced concrete columns [4,5]. Pumpable standing supports have found extensive applications in engineering practice as they are easy to transport and construct [6].

Many studies have pursued the first use of the pumpable support system in roadways of longwall faces in the United States regarding its mechanical performance [7,8,9]. Scientific research and field practice have shown that the compressive performance of the pumpable supports was related to the mechanical performance of the material filling the supports and the performance of the shell. Due to the limited rigidity and strength of the shell [7], the pumpable supports will lose their bearing capacity after the filling material reaches its peak strength [6,8,10,11].

Pumpable standing supports are generally the combination of infill material and an exterior container. The confining action of the exterior container will significantly affect the behavior of the pumpable standing support. The development of the exterior made of emerging material is always a hot topic for pumpable standing supports. A good example is the novel type of pumpable standing support developed by the University of Wollongong, for which the exterior container is made of fiber-reinforced polymer (FRP) [6,11,12]. Compared with conventional pumpable supports, this novel type of support comprises an FRP shell and cementing material as a filler. FRP has the benefits of light weight, high strength, and corrosion resistance [13,14]. FRP tubes are sufficient to provide effective confinement for the cementing material acting as a filler [15,16]. The use of high-water cementing material obtained the following benefits for the novel type of standing support: (1) given the high volume of water added (i.e., the water volume reaches up to 95%), the actual amount of solid materials consumed was minimal, which dramatically reduced the total cost [17,18]; (2) the water needed to prepare the cementing material was readily available from underground, thus reducing the extra cost otherwise incurred by long-distance transportation to the coal mine; and (3) the mixed slurry solidified quickly and had an excellent performance in compression. That is, the novel support made from such slurry adequately bore the stress and effectively controlled the roof deformation of the roadway at an early stage [19,20]. This novel type of standing support displayed significant strain-hardening features and high deformation capacity. Such standing supports shared potential technological advantages in the stability control of surrounding rocks in coal mine roadways.

Water shortages are a significant problem for coal mines in arid and semi-arid deserts or the Gobi region since preparing high-water cementing material as the support’s filler requires a large amount of water [21,22,23]. The massive consumption of water aggravated the water shortage that prevailed during this process. To overcome the above defects of FRP-confined high-water cementing material supports, we revolutionized the filling material of such supports. First, a sand-based material (SBM) was proposed by mixing the desert sand with the high-water cementing material (calcium sulfoaluminate (CSA) cement-based grouting material). Then, we studied the mechanical performance of such a support systematically. We also determined the mechanical performance of the FRP-confined SBM column under the axial load. This study used carbon fiber-reinforced polymer (CFRP) to develop the support shell.

A large number of studies have already been conducted on FRP-confined concrete columns. The results showed that a properly designed FRP confinement significantly improves the concrete’s strength and deformability [13,24]. In addition, FRP-confined concrete usually has better ductility and mitigated stiffness degradation [25,26] due to lateral constraint, which further contributed to a dramatic improvement in the anti-seismic performance of the structures [27]. However, only a few researchers have focused on the axial compression characteristics of CFRP-confined SBM columns (CSBM) [6,11,12].

In theory, CSBM have similar mechanical properties to FRP-confined high-water cementing material columns (significant strain-hardening characteristics and extensive deformability). In addition, smaller amounts of cementing material and water are consumed to prepare CSBM with a favorable cost–benefit ratio. Although current experiments mainly validated the concept of a novel type of pumpable vertical column, we are still uncertain about the axial compression characteristics of CSBM based on the limited amounts of parameters known so far. Therefore, the compressive property of CSBM needs investigation, as a comprehensive study of this novel type of pumpable vertical column, to promote its applications in underground mines in deserts and the Gobi Desert region. However, the critical issue related to the applications of CSBM is the constraint mechanism of SBM. To solve this problem, we fabricated 16 CSBM specimens for axial compression tests. The prime parameters of interest included the water–cement ratio of the cementing material, the mixing proportion of sand, and the CFRP thickness in CSBM. The research findings will enhance our understanding of CSBM performance under compressive loading. Furthermore, the present work will demonstrate the technical and economic advantages of CSBM in the surrounding rock stability control of the coal mine roadway.

## 2. Experimental Scheme

### 2.1. Specimen Details

A total of 30 specimens were prepared and tested at Xinjiang University, measuring 50 mm in diameter and 100 mm in height. First, to investigate the influence of the water-to-powder ratio, we varied the water-to-powder ratio value of the grouting material using three quantities (i.e., 1.0, 1.5, and 2.0). In addition, to evaluate the influence of the amount of sand, we varied the mixing amount of sand using three quantities (i.e., 0%, 30%, and 60%). Seven types were used as reference specimens (R series), with different water-to-powder ratios (i.e., 1.0, 1.5, and 2.0) and different mixing amounts of desert sand (i.e., 0%, 30%, and 60%). These specimens were labelled N-1.0-0 for water–powder ratio 1.0 and sand mixing 0%, etc. 

There were different types of CSBM specimens, with 2 or 3 CFRP layers, coded as C-2 and C-3, respectively. The water–powder ratio and sand mixing were selected with the same criteria as for the R series. The specimens were labeled, e.g., C-2-1.5-60 for a specimen with 2 CFRP layers, water–powder ratio 1.5, and sand mixing 60%, etc. For the test, two specimens of each N-type and C-type were measured, coded as (label)-I and -II. Table 1 gives the specimen details.

Table 2 shows the specimens grouped into four series, including the R series. Series 1 specimens had no sand mixing with a constant water–powder ratio, series 2 specimens had maximum sand mixing and changes in the water–powder ratio, and series 3 had the minimum differences in water–powder ratio and sand mixing. Finally, series 4 specimens had no sand mixing but instead variable CFRP tube thicknesses.

### 2.2. Materials Properties

#### 2.2.1. Cementing Material

Yangzhou Zhongkuang Construction New Material Technology Co., Ltd. (Yangzhou, China) supplied the CSA cement-based grouting material for this research. The cement slurry was composed of two parts, each of which had a pumping life span of over 24 h before mixing. This cement slurry had the unique advantages of being easily pumpable, having controllable solidification time, high anti-permeability performance, greenness, as well as environmental friendliness. The cement slurry was widely used in many engineering fields, such as gob-side filling, grouting reinforcement, and water plugging of the coal mines [28,29,30]. The primary hydration product of this cementing material is ettringite, which is formed by the crystallization of the tri-sulphoaluminate containing 32 water molecules. As indicated by its chemical formula (3CaO∙Al_2_O_3_∙3CaSO_4_∙32H_2_O), ettringite has many water molecules in the form of hydration products [31]. The solidified cementing material formed a dendritic network structure. Its pores are filled with free water, and a few spaces are empty [18,32].

#### 2.2.2. Sand

**Figure 1:**
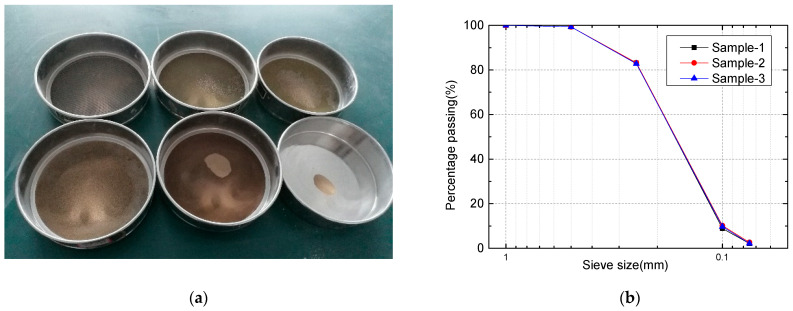
Sieving analysis of sand: (**a**) Sand after sieving; (**b**) Particle size distribution of sand.

The sand used in the tests was obtained from the Kumtag Desert of Xinjiang, China. The particle size distribution analysis was as per ASTM-C136/C136M [33]. The cumulative distribution curve of particle size (Figure 1) confirms the particle size uniformity. In addition, we observed a narrow gradation. In this study, sand improved the filler’s mechanical strength of the cementing material and maximized water’s effect to satisfy the engineering requirements.

#### 2.2.3. Sand-Based Material (SBM) 

We prepared SBM by mixing the sand, water, and cementing material together with the specified ratios. The text below details the preparation process adopted. We performed tests to determine the physical and mechanical properties of SBM for the representative mix ratios.

(1) Vicat Needle Test

**Figure 2:**
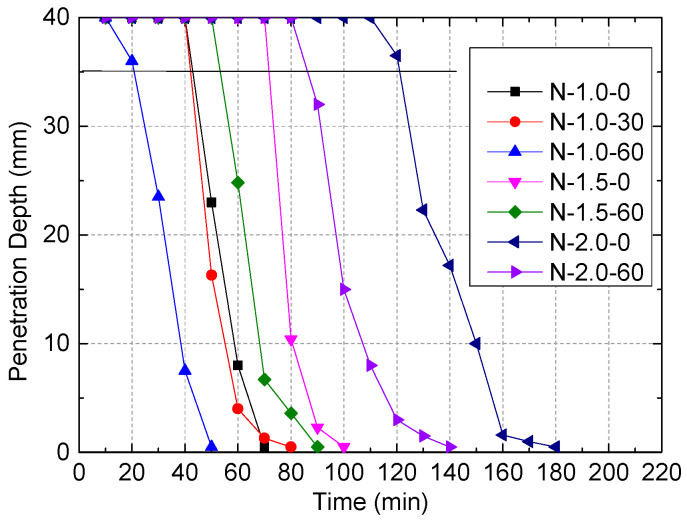
The initial and final setting times of SBM specimens.

Vicat needle test on SBM column specimens was conducted according to Standard GB/T1346-2001 [34]. We repeated each test twice and took the average of the two measurements, the results of which are given in Figure 2.

The tests results showed that as the water–powder ratio increased, the initial setting time was prolonged. However, when the mixing amount of sand increased, the initial setting time shortened. According to the established cement hydration mechanism, the initial setting time is closely related to the reaction rate, volume fraction of cement, and contact area between the cement particle surface and free water. The higher the hydration temperature, the faster the reaction rate and the shorter the initial setting time [35]. The water–powder ratio of cementitious material determined the contact area between the cement particle surface and free water and the Blaine specific surface area [36]. The higher the water-to-powder ratio, the smaller the volume fraction of cement, and the larger the distance between the cement particles, the longer the initial setting time. The sand particles added into the columns were more refined than the cement particles. As the mixing amount of sand increased, the water absorption rate increased, indirectly reducing the water–powder ratio. As a result, the initial setting time was short. According to [37], the initial setting time was considered a prime factor in determining the internal stiffness and strength of SBM. The SBM hardened within a short time through chemical reactions and thus developed certain strength features. For this reason, SBM was particularly suitable for gob-side filling, grouting reinforcement, and water plugging in coal mines.

(2) Scanning Electron Microscopy (SEM) Test

**Figure 3:**
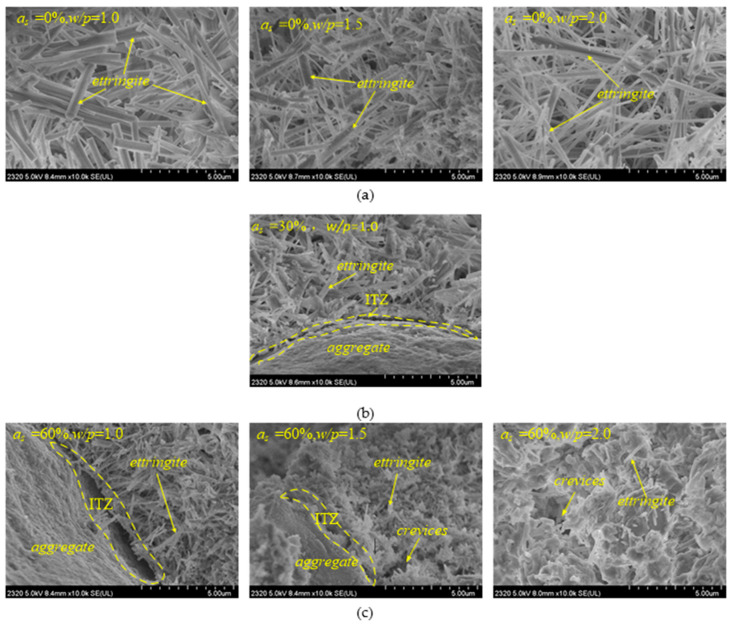
SEM images of SBM specimens: (**a**) Sand content(*a_s_*) = 0% and different water–powder (*w*/*p*) ratio; (**b**) Sand content(*a_s_*) = 30% and water–powder (*w*/*p*) ratio = 1.0; (**c**) Sand content(*a_s_*) = 60% and different water–powder (*w*/*p*) ratio.

Scanning electron microscopy (SEM) images in Figure 3 show that the ettringite thus formed presented a columnar dendritic structure on a microscopic scale, overlapping, and a staggered arrangement abounded. As the water–powder ratio increased (Figure 3a,c), the dendritic network structure of ettringite crystals became more sparse, and the ettringite columns became thinner. In addition, ettringite has a strong water absorption capacity. Therefore, the spaces in the dendritic network structure of the ettringite crystals became saturated with free water. For this reason, the CAS-based cementing material had a higher water content [18].

In addition, the mixing amount of sand influenced the dendritic network structure of the ettringite crystals, as seen in (Figure 3b,c). Under 60% mixing amount of sand (Figure 3c), the water–powder ratio increased, and the interactions between ettringite columns were insufficient to result in a compact overall structure. With the water–powder ratio being 1.0, the laps between the ettringite columns became sparser as the mixing amount of sand increased (Figure 3a–c). As a result, the integrity and uniformity of the dendritic network structure became slightly worse, and the ability to cement with the sand deteriorated.

#### 2.2.4. CFRP

All the CFRP tubes were fabricated by carbon fiber cloths and epoxy resin with a wet-layup process. The carbon fiber cloths used in the tests were from Guangdong Dianhong Information Technology Co., Ltd. (Guangdong, China). The nominal thickness of a single layer of carbon fiber cloth was 0.167 mm; the modulus of elasticity in tension was 213.21 GPa; the tensile strain at break was 1.65%. The epoxy resin was cured for seven days to achieve a tensile strength of 57.0 MPa and Young’s modulus of 2.60 GPa. Most carbon fibers (accounting for 90% of the total mass) are along the main direction (longitudinal warp). The remaining fibers (10% of the total mass) are perpendicular to it (transverse weft). The manufacturer provided the information on these basic material properties. During the CFRP tubes’ fabrication, the fibers’ main orientation (warp) direction was along the tube’s circumference, with a 60 mm overlapping zone. The tube ends were each reinforced with a 10 mm wide layer of carbon fiber belt.

**Figure 4:**
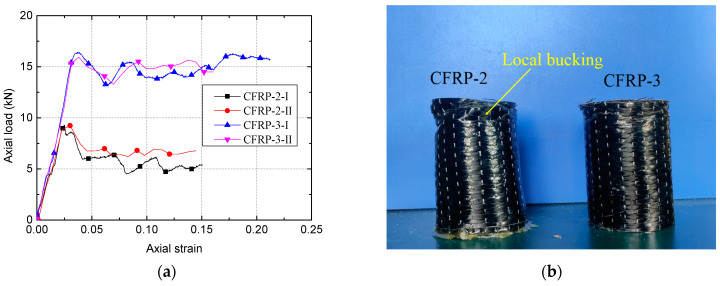
Two specimens with 2 CFRP layers (CFRP-2-I and -II) and two specimens with 3 layers (CFRP-3-I and -II) were tested: (**a**) Load–strain curve; (**b**) Failure mode of CFRP tube.

Specimens with 2 and 3 layers of CFRP and the same geometry as those specimens in Table 1, two of each type, were tested in axial compression at a constant axial deformation rate of 2.0 mm/min, and according to Standard GB/T5350-2005 [38]. As shown in Figure 4, we tested two specimens with 2 CFRP layers (CFRP-2-I and -II) and two specimens with three layers (CFRP-3-I and -II). Figure 4a shows the load–strain curve for CFRP tubes with varying thicknesses. Unlike the 2-layer specimens, the tubes reinforced with three layers of CFRP still maintained a specific bearing capacity, even after it reached the peak load (i.e., the peak load of the 2-layer specimens = 9.3 kN vs. the peak load of the 3-layer specimens = 16.3 kN). In addition, the thicker CFRP tube was less likely to buckle (Figure 4b) and had a higher strength (Figure 4a) [39].

### 2.3. Specimen Preparation

**Figure 5:**
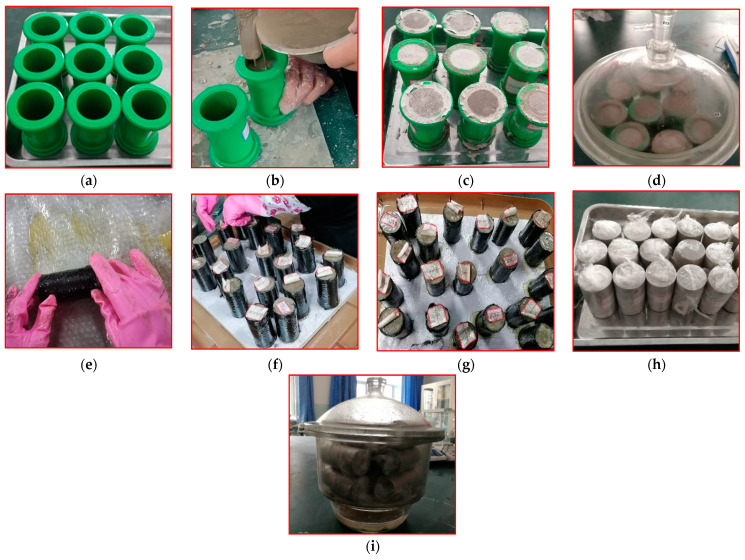
Preparation of specimens: (**a**) plastic model, (**b**) casting SBM; (**c**) curing SBM in the laboratory; (**d**) curing SBM in the dryer; (**e**) wrapping CFRP jacket; (**f**) strengthening specimens ends; (**g**) CFRP jacket curing; (**h**) covering with plastic sheet; (**i**) curing CSBM in the dryer.

**Figure 6:**
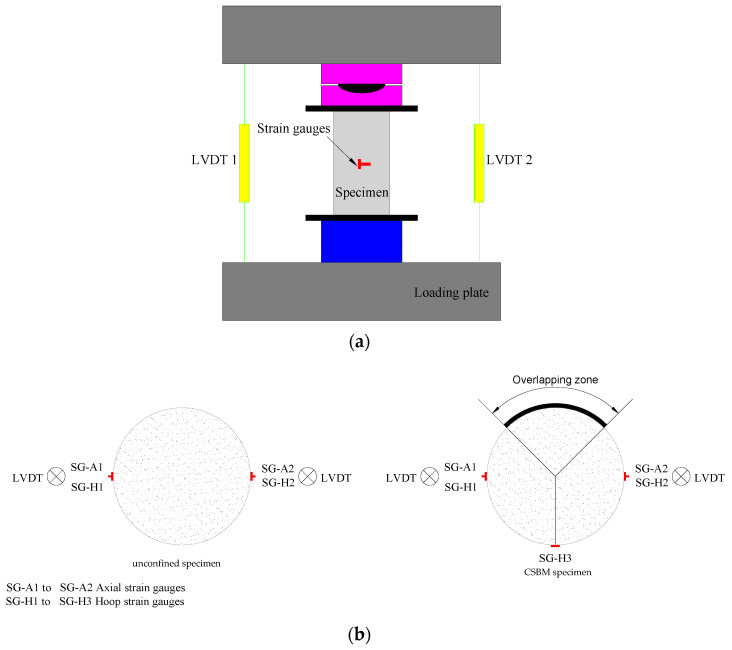
Test set-up (**a**) and layout of strain gauges and LVDTs (**b**). SG-A1 to SG-A2 represent axial strain gauges and SG-H1 to SG-H3 stand for hoop strain gauges.

We prepared the CSBM specimens in accordance with the following steps: (1) the cementing material, water, and sand were poured into a mold with specific mix ratios (Figure 5a–c); (2) after curing for 24 h, we removed the mold (Figure 5d); (3) we wrapped the CFRP on the SBM specimens with a wet-layup process (Figure 5e); (4) we adequately capped the two ends of the specimen with one layer of reinforcing CFRP (10 mm wide) (Figure 5f); (5) the CFRP-wrapped specimens were further cured in the laboratory environment for more than 24 h (Figure 5g); (6) we leveled the two ends of the specimen with a grinding machine; (7) the specimen was covered with a fresh-keeping film to prevent exposure and weathering of the filler in air. Then, the specimen was placed in a sealed container (Figure 5h,i) until the testing date (i.e., seven days).

### 2.4. Testing Configuration and Apparatus

All specimens were subjected to axial compression test on a 600 kN compression tester at Xinjiang University. As shown in Figure 6a, two linear variable differential transducers (LVDTs) were installed at the diagonal positions of the bottom loading plate to measure the overall axial deformation of the specimens. At the mid-height of the unconfined specimen, four strain gauges (SGs) were installed (Figure 6b) to measure the specimen’s axial and hoop strains. Five SGs were carefully installed in the middle of the CSBM specimen (Figure 6b). SG-A1 to SG-A2 represent axial strain gauges and SG-H1 to SG-H3 stand for hoop strain gauges (Figure 6b). Zhejiang Huangyan Testing Instrument Factory provided the strain gauges for the tests. The dimension of the sensitive grid was 80 mm × 3 mm, with the base measuring 90.5 mm × 6.7 mm (length × width). The resistance was 120 ± 1 Ω, the sensitivity factor was 2.08 ± 1%, and the strain limit was 1.0%. The loading device was calibrated before testing to ensure the accuracy and reliability of the sensor outputs of the loading device. The precision was within 1% of the measuring range. The insulation resistance between the strain gauge and the specimen was measured using an ohmmeter. The measured resistance was consistent at 120 Ω, indicating good stability of the strain gauge. ASTMD7012-2010 [40] was used to compress all specimens in the displacement loading mode with a constant rate of 2 mm/min. Test data, including strain, load, and displacement, were simultaneously recorded by the data logger.

## 3. Results and Discussion

### 3.1. Failure Mode

**Figure 7:**
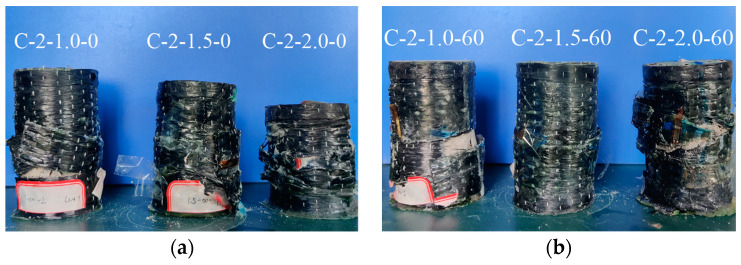

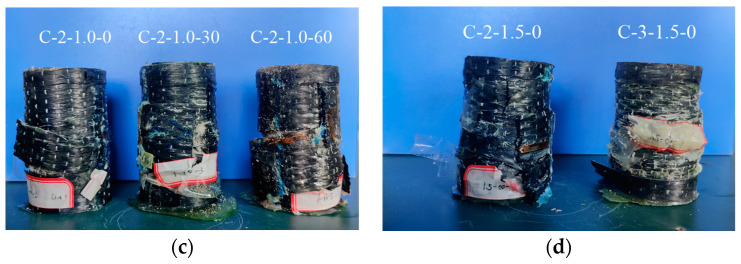
Typical failure mode of SBM specimens: (**a**) Series 1; (**b**) Series 2; (**c**) Series 3; (**d**) Series 4.

Figure 7 shows the typical failures observed in tested specimens—most of them occurred in the middle to upper positions of all CSBM specimens. Besides, the SBM specimens had considerable potential for axial deformation. The hoop fracture failure of the CFRP tubes under tensile action generated some cracking noise. These observations were slightly different from those in the CFRP-confined concrete [41].

**Figure 8:**
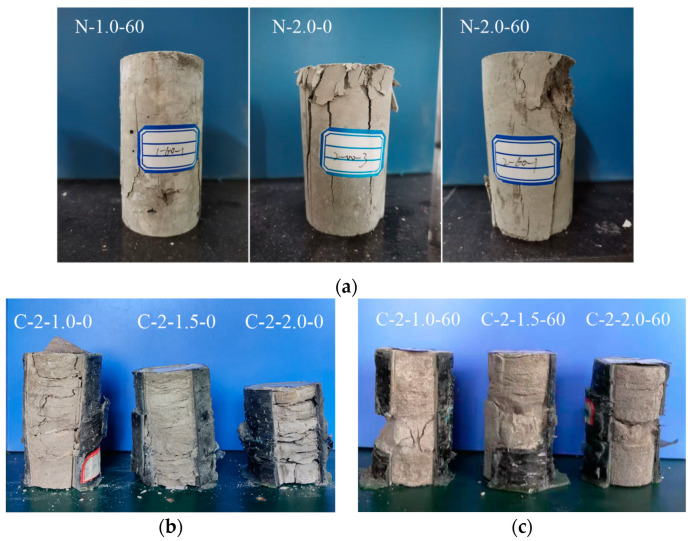

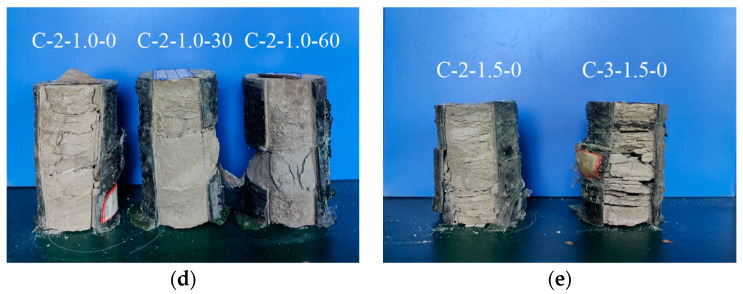
Typical failure modes of unconfined and confined cementitious grout material in SBM specimens: (**a**) Series R; (**b**) Series 1; (**c**) Series 2; (**d**) Series 3; (**e**) Series 4. R-specimens and CSBM specimens are all dry after curing in the laboratory environment for seven days.

Figure 8 shows the typical failure modes of unconfined and confined cementitious grout material in SBM specimens. With all reference specimens (R-specimens) (Figure 8a), longitudinal cracks were apparent. The cracks were nearly perpendicular, and the fracture surface was rough. That is, the R-specimens presented a compression failure and compression–shear mixed failure. The loading process did not squeeze the water out. The final state of a typical CSBM specimen after removing the CFRP shell is seen in Figure 8b–e). Contrary to other R-specimens, dominant shear failure with apparent horizontal bedding was noticed. That confirmed compression–shear failure had occurred. The higher the water-to-powder ratio (Figure 8b,c), the more significant was the appearance of horizontal bedding and the more water was squeezed during the loading process. In addition, the higher the mixing amount of sand (Figure 8d), the more sparse was the horizontal bedding and the less water was squeezed under compression. Specimens of both types, C-2-1.0-30 and C-2-1.0-60 (Figure 8d), showed only brittle cracking failure mode, but only in the first type was some water squeezed out. The thicker the CFRP tube (Figure 8e), the more significant the horizontal bedding appeared, with more water expelled during the loading process.

### 3.2. Load–Strain Behavior of CSBM

Table 3 provides the key results of CSBM specimens to facilitate the discussion, in which *P_u_* was the average ultimate load by the CSBM specimens in the ultimate limit state; *ε_u_* was the average ultimate axial strain corresponding to *P_u_*; *P_c_* was the average ultimate load from the tests of plain R-specimens; *ε_c_* was the average ultimate axial strain corresponding to *P_c_*; and *P_t_* was the ultimate load by the CFRP tube. This value was the average of the measurements from the two identical CFRP tubes under compression (Figure 4a). We supposed that *P_u_* − *P_t_* was equal to the axial load that exerted confinement on the filler of the CSBM specimen and adopted (*P_u_* − *P_t_*)/*P_c_* to evaluate the reinforcing effect of CFRP confinement on the axial load. Correspondingly, *ε_u_*/*ε_c_* represented the enhancement of the deformation ability [11]. In this study, the axial load and axial strain of the SBM specimens were positive, while the tensile stress and strain in the CFRP specimen were considered negative.

**Figure 9:**
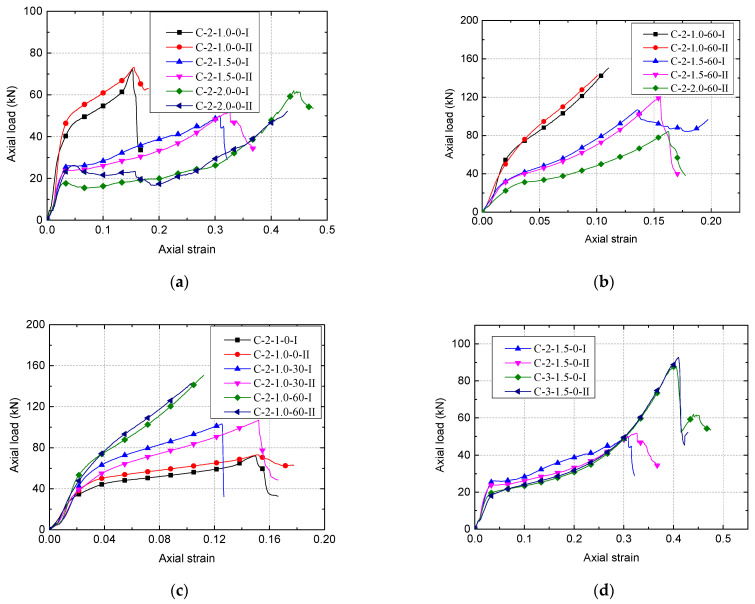
Load–strain curve of CSBM specimens: (**a**) Series 1; (**b**) Series 2; (**c**) Series 3; (**d**) Series 4.

Figure 9 shows the load–strain curve for all CSBM specimens. Note that the loading process was manually terminated when the CFRP tube was fractured. The axial strain was the average of the measurements observed from the two LVDTs. As CFRP was corrugated during the compression process, the axial strain measured by SGs was considerably different from LVDTs. Based on the suggestions proposed in existing research [6,42], we did not measure the axial strain through the SG observations in this study. As shown in Figure 9, the load–strain curves of the replicate specimens were very close to each other, indicating that the quality and representability of the two identically prepared specimens were excellent.

The load–strain curves shown in Figure 9 have two stages: an approximately linear elastic portion and a hardening portion, indicating that all the columns are well confined by the external CFRP (see also [26,41]). As shown in Figure 9, the approximately linear elastic portion has a more significant linear slope than the hardening portion. However, when the relevantly significant axial strain (i.e., 0.13, 0.20, and 0.27) (Figure 9a) was reached, the slope of the hardening portion started to increase until the ultimate state. Thus, it was apparent that the load–strain curve of specimens (Figure 9b,c) was notably separable into two phases: the quasi-linear elasticity phase (i.e., axial strain less than 0.023) and the strain-hardening phase (i.e., axial strain greater than 0.023), which was similar to the CFRP-confined concrete [26,42,43,44]. When the relevantly significant axial strain (i.e., about 0.20) was reached, the slope of the hardening portion started to increase until the ultimate state was achieved (see Figure 9d). Moreover, there is temporary but controlled load drop and load regaining in Figure 9a. This suggests that the development of adequate CFRP confining action is rather delayed (see [45,46,47]).

**Figure 10:**
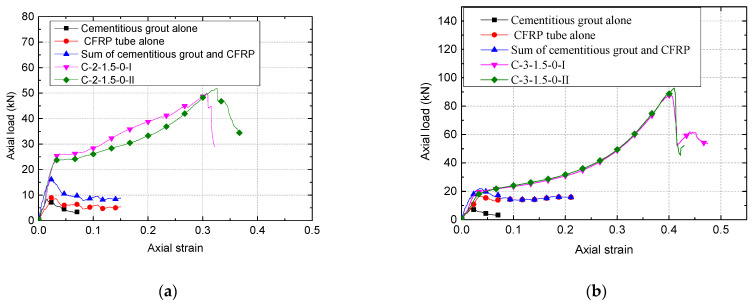
Typical load–strain curve of representative CSBM specimens: (**a**) the 2-layer case; (**b**) the 3-layer case.

To further investigate the compressive behavior of CSBM specimens, the load–strain curves of specimens from the C-2-1.5-0 and C-3-1.5-0 groups are shown in Figure 10. Herein, the load–strain curve of the cementitious grout alone was averaged from two identical R-specimens. The CFRP tube’s load–strain curve alone was directly obtained from the average value of CFRP tubes (Figure 4a) [18]. On this basis, we calculated the sum of the axial loads of the two components. It can be seen from Figure 10a that the ultimate load (i.e., 50.8 kN) and ultimate axial strain (i.e., 32.0) of the CSBM specimens were superior to those of the separate components, as expected. Compared with the CFRP-confined concrete [26,42,43,44], the direct load contribution of CFRP tubes in the CSBM specimens can be significant (Figure 10a). For design use, the direct contribution of the CFRP tube maybe not be ignored when calculating the ultimate load of the CSBM specimens. The three-layer case has similar characteristics in Figure 10b. For this reason, we supposed that *P_u_* − *P_t_* was equal to the axial load that exerted confinement on the filler of the CSBM specimen, as listed in Table 3.

### 3.3. Lateral Dilatation Behavior of Confined SBM

**Figure 11:**
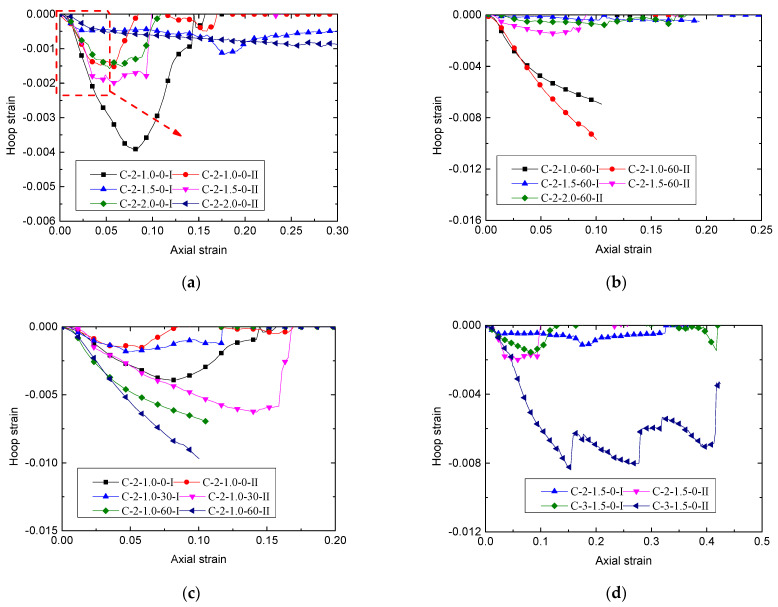
Hoop strain-axial strain curve of CSBM specimens:(**a**) Series 1; (**b**) Series 2; (**c**) Series 3; (**d**) Series 4.

The hoop strain–axial strain relationship was a prime parameter in understanding the passive confinement provided by the CFRP tube to the SBM core [41,42,43,44,45,46,47]. Figure 11 presents the hoop strain–axial strain curve of the CSBM specimens. We calculated the axial strain from the average of the two LVDT measurements and the hoop strain using the average measurements from the three externally mounted SGs that overlapped the medium height region of the specimen. At the initial stage of loading, the specimens underwent elastic deformation. The expansion deformation of the confined SBM core was small. The hoop strain of CFRP developed slowly, whereas the axial strain was in a quasi-linear relationship with the hoop strain in the CFRP. With further load increases, the confined SBM core fractured, which aggravated the lateral expansion. The CFRP confinement was activated. At this time, we heard cracking sounds produced in the cementing material. The hoop strain of the CFRP increased at a more significant rate, resulting in an enhanced slope of the curve. It was noteworthy that under continued loading, the hoop strain–axial strain curve of the specimens in the C-2-1.0-60 group (Figure 11b,c) maintained an increasing trend until the CFRP suffered a hoop fracture, which was similar to the CFRP-confined concrete [26,42,43,44]. For other CSBM specimens (Figure 11a,d), the hoop strain–axial strain curves showed a significant decreasing trend or remained stable before the CFRP suffered a hoop fracture. Meanwhile, the continued loading squeezed out free water from the CFRP tube (Figure 7a,d). Compared with the C-2-1.0-60 group, the postponed lateral dilation of the hoop strain–axial strain curves (Figure 11a,d) was related to water squeezing out of the specimens [12,18]. Moreover, the CSBM specimens had a large axial deformation under compression (Figure 9a,d). The postponed lateral dilation of the cementitious grout material attributed to the water expulsion increases the axial deformation of the CSBM specimens [12,18,42,43,44]. At the mid-height of the unconfined cylinder, four strain gauges (SGs) were installed (Figure 6b) to measure the specimen’s hoop strain. The average readings from the SGs did not accurately reflect the lateral expansion of the filler when the specimens underwent considerable axial buckling [6,18]. For this reason, we did not record the hoop fracture strain ε_h_,_rup_ of the specimen in the ultimate limit state (monitored on the cross-section at the medium height), as listed in Table 3.

### 3.4. Material Consumption

**Figure 12:**
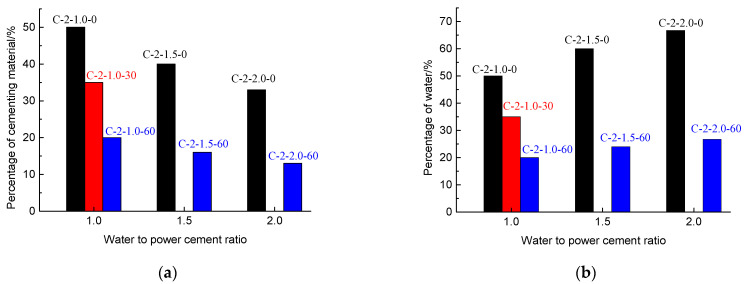
Material consumption of CSBM specimens: (**a**) the mixing amounts of cementing material with varying ratios of water to powder cement (1.0, 1.5, and 2.0); (**b**) the mixing amounts of water with varying ratios of water to powder cement (1.0, 1.5, and 2.0).

Figure 12 shows the mixing ratio of the cementing material (a) and water ratio (b) in the CSBM specimens with varying ratios of water to powder cement (1.0, 1.5, and 2.0). At the same water-to-powder ratio, the mixing amounts of cementing material and water decreased as the mixing quantity of sand increased. It is worth noting that the specimens in the C-2-1.0-0 and C-2-2.0-60 groups had a similar ultimate load (i.e., 72.6 kN vs. 82.7 kN) and ultimate axial strain (i.e., 14.2% vs. 16.5%) (see Table 3). The mixing amount of the cementing material was 50% in the specimens in the C-2-1.0-0 group vs. 13.3% in those in the C-2-2.0-60 group (Figure 12a). By increasing the mixing amount of sand, the consumption of the cementing material in the specimens in the C-2-2.0-60 group decreased by 73.4% (Figure 12a). The mixing amount of water in the specimens was 50% in the C-2-1.0-0 group vs. 26.7% in the C-2-2.0-60 group (Figure 12b). With the increase in the mixing amount of the sand, the water consumption of specimens in the C-2-2.0-60 group decreased by 46.6% (Figure 12b). Thus, increasing the mixing amount of sand improved the strength of the filler (Figure 9c) and dramatically reduced the cementing material and water consumption.

## 4. Confining Action of CFRP Composite on SBM

### 4.1. Calculation of the Confining Pressure

**Figure 13:**
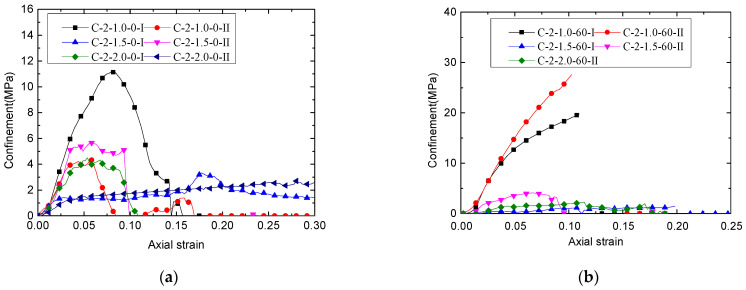

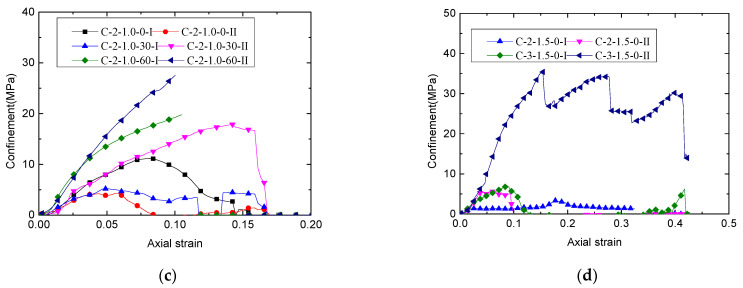
Relationship between the confining pressure–axial strain curve of CSBM specimens: (**a**) Series 1; (**b**) Series 2; (**c**) Series 3; (**d**) Series 4. The confining pressure provided by the exterior CFRP tube is theoretically calculated by substituting the hoop strain in Figure 11 into Equation (1).

The CFRP tube in the CSBM specimen interacted radially with SBM. Consequently, the CFRP tube was less likely to buckle and had a higher strength. Moreover, the presence of the CFRP tube altered the expansion features of SBM. As per above, there are already many studies concerning FRP-confined circular concrete columns. The standard constitutive models for confined concrete columns are Lam-Teng [43,48,49,50] and Samaan [51] confinement models. Jiang and Teng [43] adopted the following equation to compute the confining pressure (*σ_t_*) provided by the CFRP tube:

(1) $$\sigma_{t}=-\frac{2E_{f}t_{f}\varepsilon_{h}}{D}$$

where *D* is the diameter of the SBM core; *E_f_* is the elastic modulus of CFRP; *t_f_* is the actual thickness of the CFRP tube; and *ε_h_* is the hoop strain of the CFRP tube in Figure 11. Figure 13 shows the confining pressure provided by the outer CFRP tube, which is calculated theoretically by substituting the hoop strain of the CFRP tube in Figure 11 into the above formula.

Theoretically, the bearing characteristics of the CSBM specimens can be changed by adjusting the confining pressures of SBM [18,43,49]. According to Equation (1), the following methods were used: (1) Increasing the thickness of the CFRP tubes—Figure 9d shows that the use of thicker CFRP tubes resulted in better bearing characteristics of the CSBM specimens; (2) Using CFRP tubes with a larger elastic modulus—however, we did not evaluate this method in the present study. In addition to CFRP, the lateral expansion characteristics of SBM columns would also influence the CSBM specimens’ bearing characteristics. With other parameters being equal, the larger the lateral strain of the SBM column, the higher the confining pressure offered by CFRP would be [43]. However, some water was squeezed out of the CSBM specimens with a larger water–powder ratio (e.g., 1.5 and 2.0). As a result of water loss, the failure mode of the CSBM specimens (Figure 8b,c) was very much different from that of the other R-specimens (Figure 8a), with the latter undergoing brittle failure. As expected, the lateral expansion of the SBM column increased continuously with the axial load increase. As shown in Figure 8b and c, the larger the water–powder ratio, the smaller the CSBM specimens’ lateral deformation. In addition, the water loss significantly influenced the slope of the second section of the load–strain curve for the CSBM specimen (Figure 9a). With other parameters unchanged, the higher the mixing amount of sand, the smaller the amount of water expelled from the CSBM specimen, but the more significant the lateral strain (Figure 8c and Figure 11c) and the confining pressure (Figure 13c) would be.

### 4.2. Influence of the Water-to-Powder Ratio

As shown in Figure 13a and b, at the ascending segment of the confining pressure–axial strain curve, the confining pressure imposed by the CFRP tube decreased when the water-to-powder ratio increased. As shown in Table 3, when the mixing amount of sand was 0%, the axial load of the CSBM specimens with the water-to-powder ratio being 1.0, 1.5, and 2.0 increased by 3.37, 5.39, and 9.15 times, respectively. The ultimate axial deformation increased by 7.98-, 17.68-, and 27.19-fold, respectively. When the mixing amount of sand was 0%, the higher the water-to-powder ratio and the lower the hoop confining pressure in the CSBM specimens. By contrast, the ultimate axial deformation and the strength of the specimens increased more significantly. When the mixing amount of sand was 60%, the axial load was borne by the CSBM specimens, with the water-to-powder ratio being 1.0, 1.5, and 2.0 increased by 6.70, 7.44, and 9.01 times, respectively. The ultimate axial deformation increased by 9.24, 9.15, and 9.15 times, respectively. When the mixing amount of sand was 60%, the ultimate axial deformation and strength of the CSBM specimens with varying water-to-powder ratios increased significantly. Increasing the water-to-powder ratio did not change the increase in the deformation in the CSBM specimens. Hence, CSBM can be made with a larger water–powder ratio to achieve a more significant axial deformation while meeting the strength requirements.

### 4.3. Effect of the Mixing Amount of Sand

As shown in Figure 13c, at the ascending segment of the ultimate confining pressure–axial strain curve, the CFRP tube provided the most extensive confining pressure when the mixing amount of sand was 60% under the same axial strain. As shown in Table 3, when the water-to-powder ratio was 1.0, the axial load of the CSBM specimens with the mixing amount of sand was 0%, 30%, and 60% increased 3.37, 5.68, and 6.71 times, respectively. The ultimate axial deformation increased by 7.95, 9.16, and 9.24 times, respectively. The larger the mixing amount of sand, the more significant was the increase in the CSBM specimens’ confining pressure and strength.

### 4.4. Effect of CFRP Tube Thickness

Figure 11d and Figure 13d showed the ascending segment of the confining pressure–axial strain curve. The hoop strain of the tube reinforced with three layers of CFRP was smaller than that of the rube reinforced with two layers of CFRP at the same axial strain. The confining pressure provided by the tube reinforced with three layers of CFRP was more significant than that of the tube reinforced with two layers of CFRP. As shown in Table 3, the axial load by the CSBM specimens with two and three layers of CFRP reinforcement increased by 5.39 and 10.50 times, respectively. The ultimate axial deformation increased by 17.68- and 22.60-fold, respectively. The thicker the CFRP tube, the more significant the confining pressure was for the CSBM specimens. Moreover, the ultimate axial deformation and the strength of the specimens increased more significantly with a thicker CFRP tube. CSBM with thicker CFRP tubes achieve a more significant axial deformation while meeting the strength requirements.

Being mindful that the above discussions refer to small specimens, for CSBM specimens of larger dimensions, the adequate confining pressure exerted by SBM will change and can theoretically be evaluated using Equation (1). Before being used in the mining sites, the CSBM supports should be subject to full-scale field testing under different loading conditions (e.g., uniaxial and cyclic loading) to optimize the geometric parameters of the columns. Wu [17] and Zhang [30] suggested that the roof subsidence in a retained gob-side entry to the coal mine was a given deformation proportional to the coal seam mining thickness, typically 10–20% of the latter. For example, for a retained gob-side entry where the seam mining thickness is 3.1 m, a CSBM support built with two layers of CFRP measuring 400 mm × 3100 mm (diameter × height) is used as a gob-side backfill material. In that case, the ultimate strain of CSBM should be more than 20%. Figure 9a and d show that the ultimate strain of the CSBM specimens with larger water–powder ratios (e.g., 1.5 and 2.0) exceeded 30%. In Figure 9b, the ultimate strain of the CSBM specimens with the most considerable mixing amounts of sand (e.g., C-2-1.5-60 and C-2-2.0-60) exceeded 15%. If the ultimate strain of the CSBM support was below 20% while meeting the strength requirements, CFRP tubes with a larger water–powder ratio or a larger thickness or FRP tubes with a more significant breaking strain [41] were used to comply with the deformation requirements [52,53,54].

## 5. Conclusions

A novel column designed to maintain the stability of surrounding rock underground, termed CFRP-confined SBM column, was developed. To prove the column’s versatility, systemic experimental tests including 30 column specimens were conducted. Test parameters investigated in this research included the cementing material’s water-to-powder ratio, the mixing amount of desert sand, and the CFRP thickness of the CFRP-confined SBM column. The following conclusions have been drawn from the results of the experimental tests:

(1) The consumption of cementitious material in SBM is smaller than that of its counterparts made of pure cementitious material with similar ultimate strength, indicating that the SBM is much more cost effective;
(2) The ultimate load and ultimate axial strain of SBM are dramatically improved when it is confined by the exterior CFRP jacket;
(3) Compared with plain SBM specimens, shear failure with apparent horizontal bedding was the predominant failure mode for the CFRP-confined SBM column specimens. The higher the water-to-powder ratio, the thicker the CFRP tube, and the lower the mixing amount of sand, the more significant the horizontal bedding appeared;
(4) Using a higher water-to-powder ratio of cementitious material can result in sizeable axial deformation and a limited load-bearing capacity of the novel column;
(5) Increasing the amount of desert sand is an effective method to obtain a larger load-bearing capacity with minor axial deformation when the water–powder ratio and CFRP thickness are the same;
(6) A high confining pressure usually improves the anti-compressive performance dramatically, and thus a thicker CFRP tube is highly recommended from the design aspect.

## Figures and Tables

**Table 1:** Details of specimens.

Group	CFRP Layer	Water-to-Powder Ratio (*w/p*)	Sand Content (*a_s_*)
N-1.0-0	/	1.0	0%
N-1.0-30		1.0	30%
N-1.0-60		1.0	60%
N-1.5-0		1.5	0%
N-1.5-60		1.5	60%
N-2.0-0		2.0	0%
N-2.0-60		2.0	60%
C-2-1.0-0	2	1.0	0%
C-2-1.0-30	2	1.0	30%
C-2-1.0-60	2	1.0	60%
C-2-1.5-0	2	1.5	0%
C-2-1.5-60	2	1.5	60%
C-2-2.0-0	2	2.0	0%
C-2-2.0-60	2	2.0	60%
C-3-1.5-0	3	1.5	0%

**Table 2:** Series of specimens.

Series	Group
R	N-1.0-0
	N-1.0-30
	N-1.0-60
	N-1.5-0
	N-1.5-60
	N-2.0-0
	N-2.0-60
1	C-2-1.0-0
	C-2-1.5-0
	C-2-2.0-0
2	C-2-1.0-60
	C-2-1.5-60
	C-2-2.0-60
3	C-2-1.0-0
	C-2-1.0-30
	C-2-1.0-60
4	C-2-1.5-0
	C-3-1.5-0

**Table 3:** A summary of key results.

Series	Group	*P_u_*(kN)	*P_c_*(kN)	*P_t_*(kN)	*P_u_* − *P_t_*	*ε_u_*(%)	*ε_c_*(%)	(*P_u_* − *P_t_*)/*P_c_*	*ε_u_*/*ε_c_*
1	C-2-1.0-0	72.6	18.8	9.3	63.3	14.2	1.78	3.37	7.98
	C-2-1.5-0	50.8	7.7		41.5	32.0	1.81	5.39	17.68
	C-2-2.0-0	56.9	5.2		47.6	43.5	1.60	9.15	27.19
2	C-2-1.0-60	146.5	20.5		137.2	11.0	1.19	6.70	9.24
	C-2-1.5-60	113.4	14.0		104.1	15.0	1.64	7.44	9.15
	C-2-2.0-60	82.7	8.2		73.4	16.6	1.79	8.95	9.29
3	C-2-1.0-0	72.6	18.8		63.3	14.2	1.78	3.36	7.98
	C-2-1.0-30	104.7	16.9		95.4	14.2	1.55	5.64	9.16
	C-2-1.0-60	146.5	20.5		137.2	11.0	1.19	6.69	9.24
4	C-2-1.5-0	50.8	7.7		41.5	32.0	1.81	5.39	17.68
	C-3-1.5-0	90.2	7.7	16.3	80.9	40.9	1.81	10.50	22.60

## Data Availability

Not applicable.
